# Application of Proteomics to the Study of the Therapeutics and Pathogenicity of *Giardia duodenalis*

**DOI:** 10.3390/diagnostics12112744

**Published:** 2022-11-09

**Authors:** Ahmad Fudail Eiyad Aziz, Norhamizah Roshidi, Nurulhasanah Othman, Khayriyyah Mohd Hanafiah, Norsyahida Arifin

**Affiliations:** 1 Institute for Research in Molecular Medicine, Universiti Sains Malaysia, Gelugor 11800, Penang, Malaysia; 2 School of Biological Sciences, Universiti Sains Malaysia, Gelugor 11800, Penang, Malaysia; 3 Life Sciences, Macfarlane Burnet Institute, Melbourne, VIC 3004, Australia

**Keywords:** *Giardia duodenalis*, proteomics, vaccine and drug development, virulence, pathogenicity mechanism, PTMs

## Abstract

*Giardia duodenalis* remains a neglected tropical disease. A key feature of the sustained transmission of *Giardia* is the ability to form environmentally resistant cysts. For the last 38 years, proteomics has been utilised to study various aspects of the parasite across different life cycle stages. Thirty-one articles have been published in PubMed from 2012 to 2022 related to the proteomics of *G. duodenalis*. Currently, mass spectrometry with LC-MS/MS and MALDI-TOF/TOF has been commonly utilised in proteomic analyses of *Giardia*, which enables researchers to determine potential candidates for diagnostic biomarkers as well as vaccine and drug targets, in addition to allowing them to investigate the virulence of giardiasis, the pathogenicity mechanisms of *G. duodenalis*, and the post-translational modifications of *Giardia* proteins throughout encystation. Over the last decade, valuable information from proteomics analyses of *G. duodenalis* has been discovered in terms of the pathogenesis and virulence of *Giardia*, which may provide guidance for the development of better means with which to prevent and reduce the impacts of giardiasis. Nonetheless, there is room for improving proteomics analyses of *G. duodenalis*, since genomic sequences for additional assemblages of *Giardia* have uncovered previously unknown proteins associated with the *Giardia* proteome. Therefore, this paper aims to review the applications of proteomics for the characterisation of *G. duodenalis* pathogenicity and the discovery of novel vaccine as well as drug targets, in addition to proposing some general directions for future *Giardia* proteomic research.

## 1. Introduction

The protozoan parasite *Giardia* can cause a gastrointestinal disease known as giar-diasis, which occurs through the ingestion of cyst-contaminated food and water. It is estimated to cause 280 million cases of intestinal diseases annually worldwide, with a higher prevalence in areas with low sanitation and hygiene standards [1,2]. According to Argüello-García and Ortega-Pierres (2021), the genus *Giardia* comprises nine recognised species, with *Giardia duodenalis* (also known as *G. lamblia* and *G. intestinal*) being the only species capable of infecting a broad range of hosts, while other species only infect specific hosts. *G. duodenalis* has eight classified assemblages (A–H) [3]. Among these assemblages, only assemblages A and B are infective to humans [4]. As a result, most studies have focused on assemblages A and B of *G. duodenalis* [5]. While patients with strong immune systems usually recover within two to three days [6], in rare cases patients who are chronically immunocompromised may develop irritable bowel syndrome. Currently, laboratory diagnoses of *Giardia* spp. rely on the observation of cysts or trophozoites in stool samples, with limited options for immunological-based assays and molecular methods for the diagnosis of giardiasis [7].

*G. duodenalis* has a simple life cycle, consisting of disease-causing trophozoites and infectious cysts. Infection begins when the host ingests the cysts, which then enter the digestive tract, where they ultimately develop into motile trophozoites in the small intestine through a process called excystation. The trophozoites can then transform back into their cystic form through a process called encystation. *Giardia* can be maintained in the laboratory and induced to complete its life cycle by using laboratory methods [8], which open avenues for research on its different forms and corresponding antigens for the development of new chemotherapeutic and preventive strategies as well as a better understanding of the virulence and pathogenesis of this parasite. Among various methodological approaches with which to study this parasite, proteomics has become more widely applied due to advances in computational biology.

Although *G. duodenalis* proteomics is not a new field and genomic information on the protein-coding genes of *G. duodenalis* has been published [9], only a few of these genes have been identified as functional proteins, while the remaining proteins are still unknown and have yet to be discovered. Proteomics possesses vast potential to play a significant role in characterising proteins of the parasite in order to provide valuable information on the physiology and biology of *G. duodenalis*, complementary to genomic discoveries [10,11,12,13]. For example, despite *Giardia*’s simple life cycle, the binucleated trophozoite is able to divide without cytokinesis, producing highly infectious and adaptable cysts [14], thus rendering *G. duodenalis* encystation as a focal point of research from both genomics and proteomics perspectives. In addition to expanding proteomics to address specific inquiries on infectivity and pathogenesis, there is increasing recognition that the available proteomic characterisation of *Giardia* is mainly derived from assemblage A, while proteomic data on assemblage B remain sparse, leading to a biased understanding of this parasite’s biology [15]. In the last ten years, many proteomic studies on *G. duodenalis* have been conducted to identify molecular targets with which to block *Giardia* transmission [16], the direct effects of the expression of different gene families on the plasticity of the *Giardia* genome [17], and potential chemotherapeutic targets present in the parasite, including organelles such as ventral discs [18] or specific functional proteins [11,19,20]. Therefore, this paper aims to provide a current review of the applications of the proteomics approach in (i) identifying the protein targets for vaccine and drug development; (ii) investigating strain virulence; (iii) understanding the mechanisms of pathogenicity; and (iv) determining the post-translational modifications (PTMs) of *Giardia* proteins, summarising key findings from studies on both assemblages A and B. We further identify important areas of research with which to inform the future direction of *G. duodenalis* proteomics.

## 2. Applications of Proteomics

### 2.1. Investigating Protein Targets for Vaccine and Chemotherapy Development

Currently, there is no vaccine available to prevent giardiasis in humans, while an unrefined veterinary vaccine only reduces the symptoms and duration of cyst shedding in cats and dogs [21]. Commonly used drugs for the treatment of giardiasis, such as nitroimidazoles [22,23] and benzimidazoles, can cause mild to serious side effects, and treatment failures with metronidazole (MTZ) have been reported [22,24]. In recent years, proteomic approaches have gained popularity as a way of finding new targets to enable the development of safer effective drugs.

Several proteomic studies have aimed to investigate the biochemical aspects of the parasite [18], the significant role of proteins expressed in the completion of the life cycle [25], the antigenic surface proteins in the intestinal lumen [20], the differentially expressed proteins implicated in the mechanism of action resistance to drugs [26,27], and new potential chemotherapy agents [19,28]. Since the life cycle of *G. duodenalis* alternates between cysts and trophozoites, it is crucial to compare the proteomes of trophozoite encystment at different stages. The change in morphology, followed by the modification of protein expression levels, is vital for encystment, thus implying that the proteins involved in this process may be key targets for vaccine and drug development. 

One powerful proteomics tool with which to estimate stage-specific protein abundance is isobaric tags for relative and absolute quantitation (iTRAQ), an advanced multiplexing technique that assists in identifying and quantifying proteins simultaneously [29]. Lingdan et al. (2012) examined the differential expression of soluble proteins during *G. duodenalis* sporozoite encystation where the trophozoites and cysts were isolated from faeces. High-performance liquid chromatography (HPLC) was then used to fractionate the isobarically tagged proteins for further proteomic analyses using a database search [25]. As a result, 63 proteins were quantified by iTRAQ labelling, and these labelled proteins were then classified as cytoskeletal proteins, metabolic enzymes, cell-cycle-specific kinases, and stress resistance proteins by using MS analyses. In addition, significant differences in the expression of seven proteins in the trophozoites and cysts that are associated with encystation have also been reported in several studies [30,31]. In particular, Lingdan and his colleagues described the role of these seven proteins dissolved in the life cycle of *G. duodenalis*, raising their potential as likely targets for the development of vaccines and chemotherapies that inhibit the transmission of *G. duodenalis* into the epithelial cells of hosts [25].

Other studies have examined the potential of using repurposed drugs. Camerini et al. (2017) investigated the use of the anticancer drug 6-(7-nitro-2,1,3-benzoxadiazole-4-ylthio) hexanol (NBDHEX) to find protein targets other than phosphate dehydrogenase [19]. To identify the proteins potentially targeted by NBDHEX in *G. duodenalis* trophozoites, Camerini and her co-researchers performed a bottom-up proteomic study using a combination of SDS-PAGE, Western blot, and a mass spectrometry analysis, and detected several fluorescent protein bands in NBDHEX-treated samples, with only one or two cysteines found to be specifically NDBHEX-modified in each protein. For instance, modified Cys137 and Cys140, discovered in thioredoxin reductase, gTrxR, and Cys347, of gα-TUB structure proteins, were covalently bound to NBDHEX, suggesting that the functions of many of these protein targets were inhibited when treated with NBDHEX. The study also found that NBDHEX killed *G. duodenalis* trophozoites at a dose five times lower than that of MTZ (NBDHEX IC50:0.3 ± 0.1 mM; MTZ IC50: 1.5 ± 0.1 mM), thus supporting the idea that this drug agent could be a good option for treatment-refractory giardiasis in the future [32].

Cell surface proteins have been identified to be the source of antigens in the intestinal lumen between two genetic assemblages (A and B) of *G. duodenalis*, which may inform vaccine development. A study by Langford et al. (2002) highlighted that surface proteins might be crucial targets of protective IgA responses, and they identified several biotin-labelled proteins from total cell lysates of *G. duodenalis* WB strain (assemblage A) trophozoites and *G. duodenalis* GS/M (assemblage B) trophozoites using mass spectrometry [33]. Another protein analysis by Davids et al. (2019) led to the identification of 86 proteins in assemblage A, 51 proteins in assemblage B, and 27 proteins in both assemblages, for which 15 and 6 proteins from each group were annotated as variant surface proteins (VSPs), respectively [20,34]. A surface proteome analysis of these proteins, using a multiplex beads immunoassay, identified several conserved antigens present on the surface of the trophozoite, namely α1-giardin, α11-giardin, β-giardin, and γ-giardin, making these antigens suitable candidates for human vaccine development.

### 2.2. Investigating the Strain Virulence of G. duodenalis

Although genome sequences for assemblages A, B, and E have been published [35,36,37], little is known about the specific differences in virulence factors between *Giardia* strains and assemblages upon infection from a proteomics point of view. To the best of our knowledge, only two proteomics studies have been published that aimed to analyse the virulence proteins in *G. duodenalis*: one study investigated *G. duodenalis* in humans and cockatoos [11], and the other focused on canine isolates to understand giardiasis in dogs [38]. 

**Figure 1:**
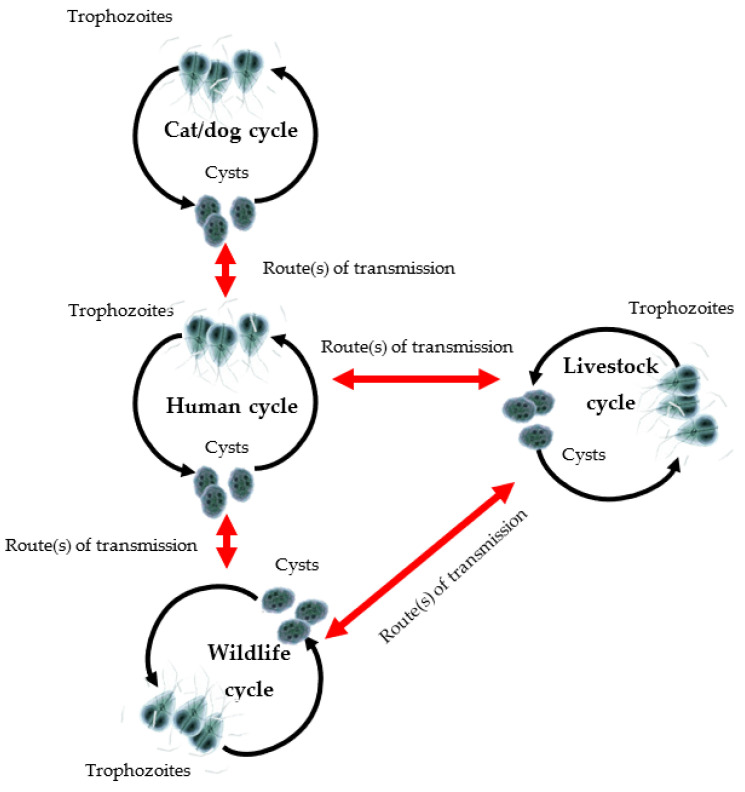
Cycles of transmission of *G. duodenalis* in mammalian hosts. Some assemblages, such as assemblage A, have low host specificity and are capable of infecting humans as well as other animals (modified from Monis et al., 2009).

The first study, by Emery et al. (2014), presented the findings of a comparative shotgun proteomic study between two different strains of assemblage A of *G. duodenalis* that may be associated with the virulence of giardiasis in mammals, namely BRIS/95/HEPU/20141 (B-2041) and BRIS/83/HEPU/106 (H-106) [11]. B-2041 and H-106 were isolated from a wild-caught cockatoo and a diarrheic child in Brisbane, Australia, respectively, the former representing a virulent strain and the latter a control strain. Since both strains were isolated from the same area from the zoonotic assemblage A1, capable of transmitting from animals to humans (Figure 1) [11,39], the authors were able to elucidate the disease mechanisms and antigenic variation of *Giardia* independent of assemblage and geographical variation. They utilised label-free shotgun proteomics by using a gel-based platform (LC-MS/MS) combined with an in-solution platform (filter-aided separation of protein; FASP) to assess the total protein abundance and proteome coverage. According to the study, 1376 proteins were identified in both strains, with a large core of 76.6% common proteins being shared between the two strains [40,41]. B-2041 was found to have a wider range of VSPs than H-106, with some of the VSPs hypothesised to be involved in giardiasis virulence. Interestingly, the authors noted that there were less antibodies specific for *Giardia* antigens in B-2041 compared to H-106, concluding that the greater the antigenic variation between different strains at the intra-assemblage level, the more diverse the population of the parasite that is capable of evading the host immune responses [11]. Indeed, the antigenic variation of *G. duodenalis* becomes a key source of variability in the virulence of different strains of the same assemblage.

Another study, by Coelho et al. (2016), analysed the proteomic mapping of soluble and insoluble protein fractions of trophozoites in canine *G. duodenalis* using 2D electrophoresis [38]. The group utilized the BHFC1 strain of *G. duodenalis*, isolated from dog stool, and identified 187 proteins, 27 of which matched hypothetical proteins, while the remaining ones had been previously annotated. Among the 27 hypothetical proteins, there were 20 soluble proteins and 4 insoluble proteins, and another 3 were found in both soluble and insoluble proteins. From the remaining 160 annotated proteins, the numbers of soluble and insoluble proteins found were 79 and 53, respectively, while another 20 were identified in both proteins. Most of the identified proteins were involved in metabolic processes, catalytic activity, nucleic acid binding, hydrolases, and oxidoreductases [38]. Additionally, some of these proteins have been related to virulence in other pathogens, namely *Candida albicans* [42], *Pseudomonas sp.* [43], and *Shigella flexneri* [44]. A comparison of the proteins of canine *G. duodenalis* with proteins of human *G. duodenalis* may lead to a better understanding of the biology of this parasite as well as of the virulence of giardiasis in different species, which can aid efforts to control zoonotic giardiasis. Notably, despite reports of high rates of *G. duodenalis* infection in domestic dogs in several countries, far fewer studies have been performed on canine isolates than on human isolates of *G. duodenalis*, highlighting a significant gap in the understanding of the risk of the transmission of giardiasis from dogs to humans [45,46].

### 2.3. Understanding the Pathogenicity Mechanism of G. duodenalis

Several studies have focused on comparing the proteome changes of *G. duodenalis* across in vitro encystation to understand the pathogenicity mechanism of *Giardia* [16,22]. A recent study, by Balan et al. (2021), formed a high-resolution quantitative proteomic analysis of encystation that covered the encystation process through to cyst maturation [16]. In their quantitative proteomics workflow, Balan et al. (2021) digested the proteins from different stages of *G. duodenalis* from its in vitro culture into peptides. The peptides were then quantified and characterized by using LC/MS/MS followed by a database search. By comparing the proteins extracted from trophozoites, low-bile primed (LB) trophozoites, and the 16 h post-induction of encystation and mature cysts, the authors identified a total of 3863 proteins across all stages [16]. In addition, 667 of these proteins had no preceding proteomic data [12,13,47,48]. The proteins identified by this group were a three-fold increase in the proteins quantified during encystation by Faso et al. (2013) [12,16]. They also determined 15, 9, 8, and 24 proteins unique to trophozoites, LB trophozoites, encystation cysts, and mature cysts, respectively. Their findings showed that each life stage of *G. duodenalis* has a significant shift in overall protein expression across encystation. For example, proteomic changes during encystation include the downregulation of cell adhesion proteins, which is linked to changes in the cytoskeleton that cause the ventral disc and flagella to disappear [14].

Increasing interest in host–parasite interactions in pathogenesis has led to the introduction of secretomic studies in *G. duodenalis*, as secretory proteins have recently been shown to play vital roles in the cross-talk between cells [49]. Mass spectrometry secretome-based profiling is a powerful strategy with which to determine and characterise the secretory proteins in the parasite, which can be based on two main proteomics workflows: in-solution digestion combined with LC-MS/MS, and in-gel digestion coupled with LC-MS/MS [10,13,50]. Duoborg et al. (2018) conducted a quantitative proteomics study on *Giardia* assemblages A and B to quantify secreted proteins, which may act as the main mediators of giardiasis pathology. In their study, the soluble and cytosolic fractions of the *Giardia* proteins were extracted from in vitro cultures of two different strains, namely the WB strain (assemblage A) and the GS strain (assemblage B). Two MS techniques were used, Q-Exactive and Orbitrap MS, to identify the proteins. The proteins were then quantified by using intensity-based absolute quantification (iBAQ). A total of 1542 GS proteins and 1641 WB proteins were identified by using Q-Exactive [10]. The authors concluded that the most abundant proteins secreted by *Giardia* are cathepsin B cysteine protease and other members of the *Giardia* family of cysteine-rich proteins. In addition, Duoborg et al. discovered a new virulence factor, *Giardia* tenascin, which contributes to a novel mechanism of *Giardia* pathogenesis and was found to be highly abundant in the whole secretome [10]. 

Similarly, other studies sought to uncover changes in the upregulation and downregulation of functional proteins, particularly in host–pathogen interactions [13,26,51]. A study by Ma’ayeh et al. (2017) characterised the excretory–secretory products (ESPs) of *G. duodenalis* during the colonisation of intestinal epithelium cells (IECs) [13]. This study reported that metabolic functions, such as glycolysis, arginine metabolism, phospholipid re-modelling, and the salvation of purines and pyrimidines, were involved as a secretory response when the trophozoites of *Giardia* interact with the IECs of a host. These results align with findings by Ringqvist et al. (2008) that *Giardia* releases glycolytic enzymes when it infects a host [52].

In addition, Ma’ayeh et al. (2017) noted competition in obtaining nutrients between the parasite and host cells, given the similarity of the metabolic proteins released by both the parasite and host [13]. For instance, enzymes such as ubiquitin-protein ligase (UPL-1) and phospholipase B (PLB) released by the parasite were upregulated as *G. duodenalis* is unable to perform de novo pyrimidine or lipid synthesis, relying solely on nutrients from the host [34,53]. A functional secretome analysis of parasite-infected IECs showed that *G. duodenalis* trophozoites initiate cytoskeletal changes as the parasite attaches to IECs very strongly, leaving marks on the cell surface and hence disturbing the arrangement of the actin cytoskeleton [54,55,56], especially the protein villin. Consequently, these findings support reports by Bhargava et al. (2015) that villin is cleaved during *Giardia* infection, severing its protective role from the actin cytoskeleton [13,57]. Collectively, the proteomic studies that focus on encystation and host–pathogen interactions have enriched the understanding of *Giardia* pathogenesis.

### 2.4. Investigating the Post-Translational Modifications of Giardia Proteins

Several post-translational modifications (PTMs) of proteins are reportedly involved in *Giardia* encystation, namely deacetylation [58] and phosphorylation [59]. Consequently, several proteomic studies have aimed to characterise the PTMs of proteins in *G. duodenalis* [4,11,15,60,61,62], such as the role of *G. duodenalis* DHHC proteins in protein S-palmitoylation during *Giardia* encystation [61]. Specifically, Merino et al. (2014) reported that nine DDHC proteins were identified in trophozoites and encysting cells of *G. duodenalis*, and concluded that the presence of DDHC proteins in the encysting parasites indicates that the protein S-palmitoylation is maintained and involved in cell signalling, protein-sorting, and protein exporting throughout encystation. However, these proteins showed variation in intracellular localization in trophozoites and patterns of cyst wall expression, suggesting that differentially regulated palmitoylation in *Giardia* encystation enables the parasite to adapt to various environments [61].

PTMs of *Giardia* proteins also cause antigenic variation, as seen in the presence of VSP subpopulations across different *Giardia* assemblages or different strains of the same assemblage [11]. Müller et al.’s (2020) attempt to characterise the surface antigens of trophozoites from three different strains of *G. duodenalis*, namely WBC6 and WBA1 (both representing assemblage A), as well as GS/M-83-H7 (classified as assemblage B), showed that VSP5 (GL50803_113793) and VSP44 (GL50803_113450) were identified in strain WBC6, VSPH7 (GSB150963) was identified in strain GS/M-83-H7, and VSPA6 (GL50803_221693), a hypothetical protein, was identified in strain WBA1 [63]. Using LFQ intensity and iTop3 protein intensities, the group found that only the WBA1 strain had the most “homogenous” trophozoites, while the others yielded mixed populations of trophozoites. 

Recently, Emery-Corbin et al. (2021) utilised a chromatin proteomics analysis, using mass spectrometry for histone identification and MaxQuant software for PTM mapping, to generate a molecular map of histone methylation, acetylation, and phosphorylation modifications in this parasite core histone [64]. The group identified over 50 sites, including sites with established roles in epigenetic regulation, amounting to a total of 56 histone modifications in *Giardia* that have been identified thus far [9,65]. Additionally, the authors were able to characterise chromatin modifiers by using protein sequence, domain, and structural homology to annotate the networks of putative histone enzymes, and identified 10 histone PTM sites detected by antibodies using immunoblots, thus providing a comprehensive and complete view of the histone PTMs in *Giardia* [64].

Another recent study, by Zhu et al. (2021), that utilised a global approach in investigating metabolic conversion mechanisms of *G. duodenalis* under stress revealed a total of 2999 lysine acetylation (Kac) sites on 956 proteins and 8877 2-hydroxyisobutyrylation (Khib) sites on 1546 proteins when *G. duodenalis* was under sugar starvation [62]. The authors noted a temporal reduction in both Kac and Khib proteins when *G. duodenalis* was cultured under sugar starvation for 72 h, indicating their involvement in energy conversion metabolism. They concluded that the correlation of acetylation and 2-hydroxyisobutyrylation expressed proteins linked to amino acid metabolism, suggesting that *Giardia*’s regulatory mechanism involves dynamic changes in acetylation modification to supply energy in the absence of glucose [62]. 

## 3. Future Directions

**Figure 2:**
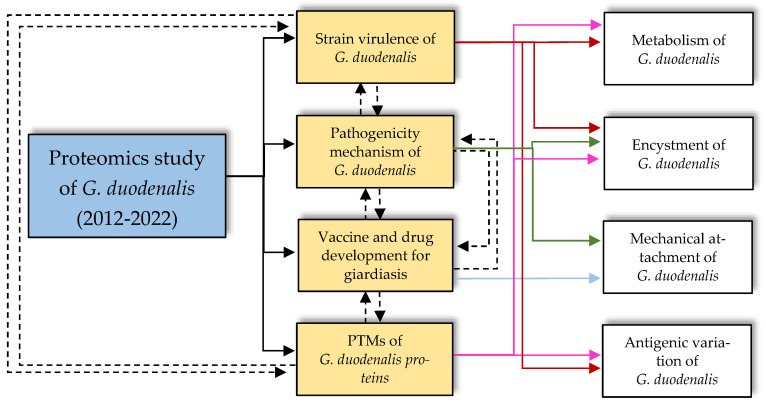

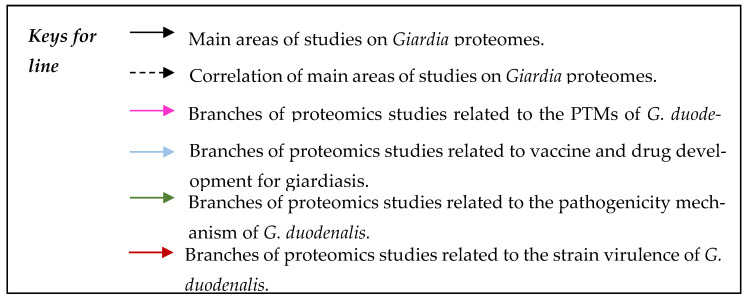
The relationships between areas of studies in the current proteomics studies on *G. duodenalis* from 2012 to 2022. The past decade of proteomics studies on *G. duodenalis* were mainly focused on determining the areas of study (as described in the gold boxes) to enhance the basic understanding of *Giardia* cellular behaviours, life cycle, and structure (as highlighted in the white boxes).

**Figure 3:**
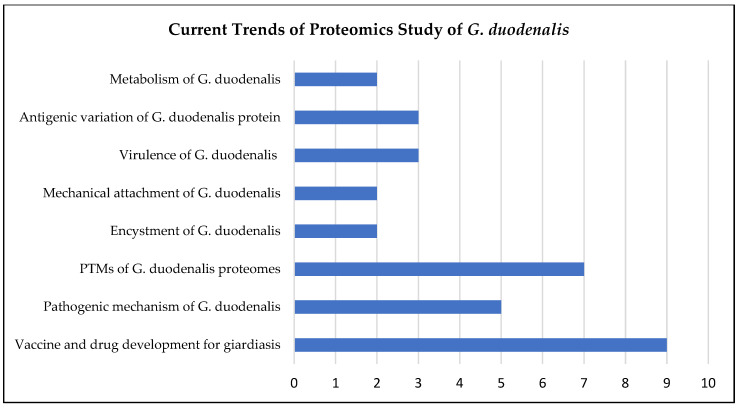
Current trends of proteomics studies on *G. duodenalis*. The numbers represent the number of studies focused on different areas of study from 2012 to 2022. The current proteomics studies on *Giardia* mainly focus on the development of vaccines and drugs for giardiasis, followed by the monitoring of the PTMs of *Giardia* proteomes.

In the last ten years, proteomics studies on *G. duodenalis* have provided significant information with which to elucidate the differential proteins expressed across the encystation of *G. duodenalis*. With the aid of advanced tools, such as iTRAQ labelling, the proteome of *G. duodenalis* cysts has been successfully investigated [25]. In addition to the process of encystation, this review found that proteomic studies of *G. duodenalis* are mainly focused on identifying novel vaccine and therapeutic targets, as well as on improving the understanding of *G. duodenalis* pathogenicity, including describing the PTMs of *Giardia* proteomes, as illustrated in Figure 2 and Figure 3 and summarised in Table 1. 

In the future, proteomic research may focus on comparing soluble and insoluble proteins extracted from cysts and trophozoites, which may enable the discovery of common proteins in soluble and insoluble fractions of both cysts and trophozoites that could become key chemotherapy targets for giardiasis. Proteomics can be further utilised to identify different protein sites that could be targeted by newly discovered drug agents [66,67] to determine whether they may be useful as novel treatments of giardiasis. In particular, proteomics has the potential to be a very beneficial tool for identifying protein targets of the virulence, zoonotic transmission, and host specificity of various *Giardia* assemblages, beyond the human-infective assemblages A and B. Through these inquiries, new insights into inter- and intra-assemblage variations and their association with evolutionary origins, biology, and protein function can be gained. Nevertheless, genomic characterisation needs to be developed first to allow the future comparative proteomic analyses of these isolates to move forward [63]. Future proteomic studies on *G. duodenalis* may also focus on investigating the post-transcriptional regulation of this parasite, especially on translational inhibition relating to *Giardia*’s Pumilio homology proteins (Puf), which may have a crucial role in the encystation of this parasite [68]. With the completion of a comprehensive molecular map of histone modifications in *Giardia* by Emery-Corbin et al. (2021) [64], chromatin proteomics may achieve the mapping of previously reported non-histone proteins, such as tubulin [69] and cyclin B [70], by using quantitative MS profiling to confirm their dynamic variations in regulation during parasite development. Ideally, proteomics research should be linked with experimental approaches to move beyond protein identification and progress to the in vitro or in vivo investigation of these proteins as players in pathogenesis, virulence, and metabolism, and later to the validation of their role as biomarkers or drug and vaccine targets that can lead to new tools with which to control giardiasis.

## 4. Potential and Limitations of Current Proteomics Studies in Biomedical Research

The PTMs of *Giardia* proteins contribute significantly to the parasite’s strain virulence and its pathogenicity mechanism. The PTMs in the parasite expand the protein functionality and diversity, which allows *G. duodenalis* to acquire an elusive mechanism with which to bypass host defences and successfully complete its life cycle in a host. Currently, some transcriptomics studies on *G. duodenalis* have elucidated the expression of a few virulence factors during the interaction, such as high-cysteine membrane proteins [71] and pro-inflammatory cytokines [72]. Henceforth, a proteomics approach can potentially investigate the role of these proteins expressed during the host–*Giardia* interaction, as these proteins may damage the epithelial cells of a host and contribute to an evasive bypass of a host’s immune system. Since proteomics enables the comparison of proteins between healthy individuals and those infected with *Giardia*, this approach also has significant potential to enlighten researchers about effective disease management for giardiasis. Although *Giardia* proteomics is currently limited to enhancing the basic knowledge of the parasite proteome and host–parasite interaction, extending proteomics for clinical applications may someday aid in the direct management of giardiasis.

A key limitation in proteomics research on *Giardia* is a remaining paucity of information on assemblages that infect species other than humans. Nevertheless, advancements in the characterisation of proteomes of most assemblage A and B strains, and a few assemblage E strains, raises the potential to elucidate proteomes of other assemblages in the future, given that a strong fundamental proteomics workflow with which to investigate *Giardia* has now been established. However, proteomic studies on *Giardia* assemblages other than A, B, and E remain of limited interest, because these assemblages are not infective to humans, there is sparse genomic data for reference, and the cost of downstream processing is prohibitive. This lack of understanding of other assemblages constitutes a prevailing gap in the understanding of *Giardia* as a whole, which may have implications for current efforts to understand how the human-infective parasite interacts with hosts.

## 5. Conclusions

This review summarises the importance and applications of proteomic studies in studying giardiasis. Proteomics has been successfully applied to study the parasite across varying lines of inquiry, such as determining protein targets for vaccine and drug development, investigating the virulence of giardiasis, understanding the pathogenic mechanism of *G. duodenalis*, and investigating the PTMs of *Giardia* proteins, findings from each of which inform the others. Current proteomics studies on *Giardia* are still limited because the genomic sequences are not well-established in all eight assemblages of the parasite. Even with this being the case, we can foresee that the integration of proteomics technology and transcriptomics will enable the identification of novel genes in the parasite that are overlooked by gene prediction programs. In the near future, advanced technologies such as MALDI-TOF, HPLC, and iTRAQ labelling techniques may be used more frequently. In conclusion, even with the limited and emerging published genomic information to date, the existing literature undoubtedly reveals the potential of proteomics to significantly enrich the understanding of the parasite and shine light on new avenues of research for the effective control of giardiasis.

## Figures and Tables

**Table 1:** Summary of proteomics-based analyses of *G. duodenalis* from 2012 to 2022.

No.	Purpose of Proteomics Study	Approach	Subtypes of *G. duodenalis*		Target Protein	Database	Software Programme	Remarks/Advantages	References
			Assemblage	Strain					
1.	Mechanical attachment of *G. duodenalis*	1D-sodium dodecyl-sulphate polyacrylamide gel electrophoresis (SDS PAGE), 2D-SDS PAGE, nano liquid chromatography-tandem mass spectrometry (nLC-MS/MS) and MALDI-TOF/TOF MS	A	WBC6 strain	Ventral disc	NCBI-nr	MASCOT	Identification of a ventral disc that may be associated with parasite adhesion to the intestinal epithelial cells (IECs). Suggests a possible target for developing a giardiasis vaccine that can also be found using the proteomics approach.	[18]
2.	Encystment of *G. duodenalis*	iTRAQ labelling, SFX fractionation, and reversed-phase nano (RPLC-MS/MS)	D	Changchun strain	Soluble proteins from trophozoites and cysts	GiardiaDB	MASCOT	Elucidation of the roles that specific proteins play in the parasite's life cycle through a comparison of the proteomes of trophozoite encystment at different stages.	[25]
3.	Evaluation of drug treatments/vaccine constructions on *G. duodenalis*	2D-PAGE, isoelectrofocusing (IEF), and LC-MS/MS	A	WB strain	Albendazole (ABZ)-resistant and -susceptible trophozoites	NCB-nr	MASCOT v1.6b9	First report of proteomes of the ABZ-sensitive and -resistant *G. duodenalis* clones. Modes of action of the drugs and their resistance mechanisms in the parasite through differentially expressed enzymes involved in the glycolytic and arginine dihyrolase pathways determined using proteomic analysis.	[27]
4.		2D-PAGE and MALDI-TOF MS	A	WBC6 strain	Cytoskeleton proteins in *Giardia* trophozoites	NCBI-nr	MASCOT v2.3	Giardicidal activity of drug compounds on the parasite’s structure.	[28]
5.		1D-NuPAGE 4-12%, Western blot analysis, and LC-MS/MS	A	WBC6 strain	Proteins in *Giardia* trophozoites	GiardiaDB, UniProt, Protein Data Bank (PDB)	ELM, BLASTp MutAlin, SwissModel, I-TASSER, HHpred and Phyre2 PsiPred, and Disopred3	Identification of unknown drug targets in the parasite led to the discovery of a new mechanisms associated with the drugs’ cytotoxicity in the *Giardia* trophozoites.	[19]
6.		Western blot analysis, streptavidin affinity chromatography, SDS-PAGE, and high-pressure LC-MS/MS (HPLC-MS/MS)	A	WBC6 strain	Surface proteins of *Giardia* trophozoites	GiardiaDB	ProteinPilot v2.0, SignalP server v3.0, and CSS-Palm	Identification of proteins recognised by immune serum reveals the array of conserved antigen candidates, which can overcome the rate-limiting step in the construction and design of a giardiasis vaccine.	[20]
			B	GS/M strain					
7.		nLC-MS/MS	A1	C4 strain	Nitazoxanide (NTZ)- and metronidazole (MTZ)-resistant trophozoites	GiardiaDB	MaxQuant v1.5	Comparison of protein levels between wild-type and drug-resistant strains. Variability of the trophozoite population in *G. duodenalis* reported.	[26]
				1062ID10 strain					
				713M3 strain					
8.	Virulence of *G. duodenalis*	1D-SDS PAGE, nLC-MS/MS, filter-aided sample preparation (FASP), and gas-phase fractionation (GPF)	A1	BRIS/95/HEPU/2041	Variant surface proteins (VSPs)	GiardiaDB	Global proteome machine (GPM) and X1Tandem	The first comparative and quantitative proteomic analysis of *G. duodenalis*. Profile of protein abundance in different strains of *G. duodenalis*.	[11]
				BRIS/83/HEPU/106					
9.		2D-SDS PAGE and MALDI-TOF MS	A	BHF1C strain	Soluble and insoluble proteins in trophozoites	GiardiaDB	MASCOT v2.1, Protein Blast, and Panther	Protein mapping in trophozoites of each strain. Proteins associated with metabolism and the virulence of *G. duodenalis* identified. Characterisation of the proteomes of *G. duodenalis* isolated from different organisms such as dogs, humans, and cats.	[38]
				Portland-1 strain					
10.	Pathogenicity mechanism of *G. duodenalis*	nLC-MS/MS, ELISA, SDS-PAGE, and Western blot analysis	A	WBC6 strain	Excretory–secretory products (ESPs) of *Giardia* trophozoites	GiardiaDB	Proteome Discoverer v1.4 and MudPIT	First identification of *Giardia* ESPs and their effects on intestinal epithelium cells (IECs). Characterisation of secretome when the parasite interacts with the host’s cells.	[13]
			B	GSH7 strain					
11.		LC-MS/MS	A	WB-1B strain	Trophozoites, low-bile primed trophozoites, and the 16 h post-induction of encystation and mature cysts	UniProt, PFAM	MaxQuant v1.5.8.3, STRING v11, I-TASSER, and Auto Dock Tools	The most extensive analysis of encystation in *Giardia.* Changes in secretory machinery during encystation and bivalent switching within metabolisms described.	[16]
12.	Post-translational modifications (PTMs) of *Giardia* proteins	1D NuPAGE 4-12% and rpLC-MS/MS	A	WBC6 strain	14-3-3 proteins	PDB	Program Phaser, COOT, Refmac5, PyMOL, secondary structure matching (Superpose), and NAMD v2.8	Structural proteomics analysis of *Giardia* showing common features of select proteins with other protein family members. Elucidation of the structure and function of PTMs in *Giardia* 14-3-3 proteins, which could be potential drug targets for giardiasis.	[60]
13.		SDS-PAGE, Western blot, chloroform/methanol extraction, and flow cytometer	A	WBC1267 strain	DHHC proteins (also known as protein acyltransferases) in the trophozoites and encysting cells of *Giardia*	Ensembl, NCBI, PFAM, GiardiaDB and UniProt	CD-HIT, PROMALS3D, ProtTest, CSS-Palm v3.0, HMMTOP, SMART, TMHMM, TMPred, and Block Mapping and Gathering with Entropy (BMGE)	Proteomic analysis of *Giardia*, highlighting the relationship between the PTMs of *Giardia* proteins and the parasite’s encystation.	[61]
14.		Western blot analysis, SDS-PAGE and nLC-MS/MS	A1	WB-M3 strain	MTZ-resistant and -susceptible trophozoites	GiardiaDB, UniProt and PRIDE	Search Tool for the Retrieval of Interacting Genes (STRING), Xcalibur, Proteome Discoverer v1.3, MASCOT, and DAVID bioinformatics resource	Identification of differentially expressed proteins from the dynamic changes in a wide range of PTMs between MTZ-resistant and -susceptible *Giardia* lines. This study is the first reported proteome of MTZ-resistant and -susceptible parasite pathogens.	[15]
				BRIS/83/HEPU/106-2ID10					
				BRIS/83/HEPU/713-M3					
15.		Trifluoroacetic acid (TFA) extraction and LC-MS/MS	A	WBC6 strain	VSPs in *G. duodenalis* trophozoites	GiardiaDB	MaxQuant v1.5.4.1	Determination of the expression levels of predominant VSPs in different isolates. Comparison of differentially expressed proteins in *G. duodenalis* trophozoites in different strains.	[63]
				WBA1 strain					
			B	GS/M-83-H7 strain					
16.		Acetone precipitation, SDS-PAGE, and nLC-MS/MS	A	WB-1B strain	Histone proteins	GiardiaDBand UniProt	MaxQuant v1.5.8.3, PRIDE, CLUSTALW and Aline programme v1.0.025	First complete and comprehensive view of *Giardia* histone PTM. Mapping of *Giardia* protein PTM sites associated with the epigenetic regulation of the parasite.	[64]
17.		Reverse-phase high-performance liquid chromatography (rpHPLC), SDS-PAGE, and iTRAQ labelling	A	WBC2 strain	Trophozoite of *G. duodenalis* under sugar starvation	UniProt, CORUM, and STRING v10	MaxQuant, R Project, and Cytoscape v3.2.1	Analysis of *Giardia* protein PTMs in the energy–metabolism conversion that occurs in the parasite and further provides significant information on evolution from prokaryote to eukaryote.	[62]

## Data Availability

Not applicable.
